# Implementation and Validation of a Limiting Component Quantification Method for qPCR

**DOI:** 10.3390/ijms27062717

**Published:** 2026-03-16

**Authors:** Andreas Untergasser, Quinn D. Gunst, Vladimir Benes, Maurice J. B. van den Hoff

**Affiliations:** 1 Zentrum für Molekulare Biologie der Universität Heidelberg, Im Neuenheimer Feld 329, D-69120 Heidelberg, Germany; 2 European Molecular Biology Laboratory (EMBL), Genomics Core Facility, D-69117 Heidelberg, Germany; benes@embl.de; 3 Department Medical Biology, Amsterdam University Medical Centers, Location AMC, Meibergdreef 15, 110AZ Amsterdam, The Netherlandsm.j.vandenhoff@amsterdamumc.nl (M.J.B.v.d.H.)

**Keywords:** qPCR, RDML, RDML-Tools, optical calibrators, SYBR Green I, EvaGreen, hydrolysis probe, absolute quantification, relative quantification, Ncopy

## Abstract

Quantitative polymerase chain reaction (qPCR) is a widespread method to quantify RNA or DNA. The results are reported as cycle of quantification (Cq), scaled to absolute numbers of copies or relative to reference genes. The reported Cq values of the same reaction vary between different machines and cannot be compared between different laboratories. This study shows that the third derivative zero (TD0) method is machine independent and more reproducible than the classic Cq calculations. Together with the mean PCR efficiency it allows the calculation of the number of copies initially present (Ncopy), a parameter easy to interpret. A large dataset was created for the evaluation of this method including amplicons with different length, primer concentrations, reaction mixes, and fluorescence reporter systems. Furthermore, the calculated Ncopy values can be corrected at the same time using known concentrations of a standard and for the expression of reference genes and combining absolute and relative quantification. The algorithms were implemented in the open-source program RDML-Tools, which can perform all steps of a qPCR analysis using the raw fluorescence amplification data and is available on the internet. We conclude that qPCR analysis today should widen its focus and include the three essential parameters, TD0, mean PCR efficiency and Ncopy.

## 1. Introduction

Quantitative polymerase chain reaction (qPCR), in the past sometimes referred to as real-time PCR, is a method to detect DNA and, with a preceding reverse transcriptase step, also RNA [1,2,3,4]. Provided that the assay conditions are carefully optimized, qPCR is highly specific and covers a very wide dynamic range, allowing the detection and quantification from a single up to billions of target molecules in an individual reaction. In the last decades it has evolved to a standard method in molecular biology laboratories, and it is supported by industry, providing a large range of qPCR machines and commercial reaction mixes and kits. Today, qPCR is considered a mature technology and data are reported with little consideration.

Many qPCR machines are available on the market that either use 96 or 384 well plates, allowing the processing of 20–50 µL or 5–20 µL reaction volumes, respectively. qPCR combines the exponential amplification of a DNA target with continuous monitoring of the increasing DNA concentration. The DNA concentration is measured by including either fluorescent DNA binding dyes or fluorescently labelled probes, like hybridization or hydrolysis probes, in the reaction mix [5]. The fluorescence of the DNA binding dyes, like the commonly used non-saturating dye SYBR Green I or the saturating dye EvaGreen, increases upon sequence-unspecific binding to the DNA. Fluorescently labeled probes are short single stranded oligonucleotides with a fluorochrome and a quencher attached. Hydrolysis probes are designed to bind a specific sequence in between the primers and the polymerase hydrolyzes the probe in the amplification step. The hydrolyzation releases the fluorochrome from the quencher and thereby becomes fluorescent. The fluorescence of each reaction is measured optically at the end of each amplification cycle. Hybridization probes have a comparable structure but are designed such that upon hybridization of the probe to its target, the fluorochrome becomes fluorescent. In this case the fluorescence is measured at the end of the annealing phase. In all cases the PCR machine stores the raw fluorescence data from which the background signal is subtracted per cycle. It should at this point be noted that all machines still have their own storage format [6]. While all machines record a twice as high fluorescence value for a sample with twice the amount of DNA, the scaling of the fluorescence values may differ between machines by a factor 1000. Within the exponential phase of the PCR, the reaction can be mathematically described with the formula:

(1) $$N_{n}=N_{0}\times E^{n}$$

Ideally, the DNA doubles in each cycle, which would correspond to a PCR efficiency (E) of 2 as the base of the exponential function (equal to 100%). With a given number of copies at the start of the qPCR (N_0_), the number of copies (N_n_) at the n^th^ cycle (n) can be calculated (Equation (1)).

**Figure 1:**
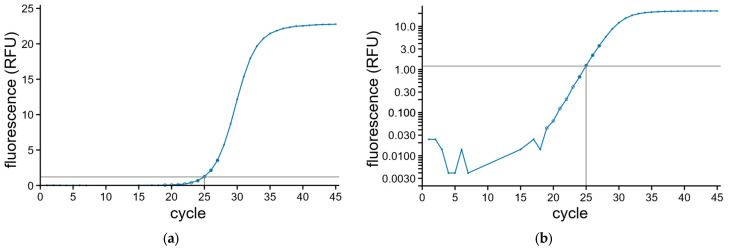
Both figures show the identical, baseline corrected amplification curve. Big dots mark the exponential phase. The threshold and the called Cq value are indicated by grey lines: (**a**) The fluorescence is plotted on a linear scale; (**b**) The fluorescence is plotted on a logarithmic scale. In the logarithmic plot the exponential phase is identified as a straight line.

When the measured raw fluorescence data is plotted on a linear scale as a function of the respective amplification cycle, the obtained curve has a typical S-shape (Figure 1a). The different phases during the PCR can be better appreciated when plotted on a logarithmic fluorescence scale (Figure 1b). There are four distinct phases. It starts with the ground phase, in which the DNA is exponentially amplified, but the fluorescence of the small amount of produced DNA is so low that it does not surpass the ground phase fluorescence, which is due to the consumables, the reaction components and in case of DNA-binding dyes, non-amplified double-stranded DNA. At some point the exponentially amplified DNA reaches detectable amounts and passes background fluorescence. This phase is called the exponential phase and easily identified as the straight part in the amplification curve when plotted on a logarithmic scale. When one of the components of the reaction mix, usually the primers or SYBR Green I, becomes limiting the exponential increase in fluorescence stops and the exponential phase ends by entering in the transition phase towards the plateau phase. The point where the exponential phase has ended is dubbed the second derivative maximum (SDM).

Most often the first step of data analysis is carried out using the machine software. The baseline fluorescence is calculated, usually by averaging the fluorescence of the first cycles, sometimes up to cycle 15, of each individual reaction and subtracted from all measurements of the respective reaction. Alternatively, the machine software fits a straight line through the fluorescence of the first cycles and uses this line to correct the fluorescence of each amplification cycle. Next, a threshold fluorescence level is defined by the machine or the operator within the exponential phase for the entire run [3]. The crossing point of the background corrected amplification curve with the threshold is determined and the fractional cycle is calculated for each reaction (Figure 1). This value is called the cycle of quantification (Cq). The lower the number of target copies at the start of the PCR in the reaction mix is, the more amplification cycles are required to reach this threshold level. Therefore, low DNA concentrations at the start of the reaction result in high Cq values and high DNA concentrations in low Cq values.

The Cq values are commonly used for absolute or relative quantification. For absolute quantification a calibration curve is prepared by diluting a DNA standard of known concentration or number of copies and measured for each target in the qPCR. A calibration curve is constructed by plotting the Cq value of each reaction to its respective input. Through these data a straight line is fitted using linear regression. The slope of the line is a measure for the PCR efficiency, and this calibration curve is used to convert the Cq values of the unknown samples to a concentration or number of copies. This type of quantification is named absolute quantification [7]. Today, digital PCR (dPCR) offers a direct approach to absolute quantification by fractioning the reaction mix into small droplets and using Poisson statistics for accurate quantification [8,9,10]. An easier type of quantification is referred to as relative quantification. In relative quantification a set of validated reference genes, which have a constant expression level throughout the experiment [11], are used to correct the measured Cq values using the ΔCq method [7]. The results of the latter method are very biased, because almost never the target doubles in every cycle during the PCR. To remedy this, the efficiency-corrected Cq value was introduced in a seminal paper by Pfaffl in 2001 [12]. In this method the PCR efficiency is determined using a standard curve, a variant of the calibration curve of which only the slope is used to calculate the efficiency. However, in practice, the PCR efficiency is not determined and as a consequence, differences in the PCR efficiency between different targets are not corrected in the results. Moreover, the PCR efficiency for different targets of interest as well as for reference genes can differ largely. This omission can produce largely biased or even meaningless results [13]. A far easier method was introduced by LinRegPCR using the threshold value (Nq), the Cq, the PCR efficiency (E) and a rearrangement of the kinetics equation for qPCR (Equations (1) and (2)) to calculate the starting concentration (N_0_; Equation (2)) [14,15].

(2) $$N_{0}=\frac{N_{q}}{E^{C_{q}}}$$

The COVID-19 pandemic has univocally unveiled the need for better and standardized reporting of relevant qPCR data. The results of the COVID qPCR assays were reported worldwide as Cq values and it quickly became evident that such Cq values could not be compared between different countries, between different laboratories within a single country or even between different qPCR machines in the same institute [16]. Even though standard assays were used, differences in hardware and analysis software as well as non-standardized setting of the ground phase, of the background subtraction method, setting of the quantification threshold and differences in PCR efficiency resulted in Cq values that cannot be compared and accurately interpreted.

In this study we present a simple and robust implementation in RDML-Tools of a new method to determine the number of copies of target molecules in a PCR. It is referred to as a limiting component method and reports the number of target copies at the start of the reaction (Ncopy). This method was presented on the 10th Gene Quantification Event 2023 in Freising, Germany [17] and a theoretical approach modeling the reaction components is further discussed in this special issue [18]. For this study we use an empirical approach and present experimental data to validate this theoretical model. Furthermore, this method allows us to combine absolute and relative quantification. With the implementation of this method in RDML-Tools, this new version provides a qPCR data analysis pipeline encompassing the import of fluorescence data into RDML-Tools, analysis of melting curves and amplification curves, determination of Ncopy per reaction, inter-run correction, reference gene validation, normalization using reference genes, calibration of Ncopy with a single standard, between-group statistics and finally graphical presentation of the analysis results. Using this pipeline, qPCR results are reported in the number of targets at the start of the reaction (Ncopy) that is comparable between different machines, laboratories and countries.

## 2. Results

### 2.1. TD0 Is Superior to Cq

Usually, a Cq value is calculated by setting a threshold fluorescence in the exponential phase and calculating the fractional cycle required to reach this threshold for each reaction [19]. This approach, although widely applied, has two major disadvantages. Firstly, the optical sensitivity of the machine and the measured ground phase noise resulting from both the background of the hardware of the machine and the assay, determine the start and the length of the exponential phase. Secondly, the placement of the quantification threshold is different for different machines and can even be user adjusted. Its position directly influences the determined Cq value; the higher the threshold is set, the higher the Cq values will be for all reactions.

The LightCycler machines from Roche determine the second derivative maximum (SDM) of the amplification curve by curve fitting [19,20]. The SDM is reached when the exponential phase has ended due to the fact that one of the reaction components has become limiting. The advantage of using the SDM is that it does not require setting a fluorescence threshold.

A mathematical advantage of calculating derivatives is that any constant baseline fluorescence is lost when the first derivative is determined and any linear baseline when the second derivative is determined. This makes the SDM calculations insensitive to baseline corrections applied by the machines. Mathematically, a second derivative minimum or maximum results in a zero crossing of the third derivative at the same position. In the implementation in RDML-Tools, the zero crossing of the third derivative within two cycles to the first derivative maximum is used. As with each derivative the impact of the measurement noise increases, the second derivative is smoothed by calculating a moving geometric mean of three cycles before calculating the third derivative and determining the zero crossing, which is referred to as TD0.

To evaluate the results of the TD0 calculation one large reaction mix was prepared to amplify the published 259 bp Follistatin-like 1 (FSTL_1_*_259) amplicon of exon 7 [21] using 0.5 ng/µL human genomic DNA, pipetted into the plate in different volumes (Table 1) and processed in three different machines. The plates for the Thermo Fisher Scientific QuantStudio 6 (Thermo Fisher Scientific Inc., Santa Clara, CA, USA) and StepOne (Thermo Fisher Scientific Inc., Santa Clara, CA, USA) were pipetted from a single master reaction mix and ran at the same time side by side in Heidelberg (Germany). The reaction mix for the Roche LightCycler 480 II (Roche Diagnostics, Basel, Switzerland) was prepared and run in Amsterdam (The Netherlands) using the same Roche SYBR Green I master mix stock (Roche Diagnostics, Basel, Switzerland), the same primer stock and the same genomic DNA solution as in Heidelberg. On all three machines the same cycling protocol was used: an initial 10 min 95 °C step followed by 50 cycles of 15 s at 95 °C, 20 s at 60 °C and 45 s at 72 °C.

Table 1 summarizes the results of the PCRs in which the concentration of the reaction components in each reaction mix is the same, despite the differences in volumes. In this set up, one would expect the same Cq value in each reaction. It comes as no surprise that the Cq values automatically called by the machines, without any further user optimization, differ between the Roche LightCycler 480 II and Thermo Fisher Scientific QuantStudio 6, though being reasonably close to each other. The Thermo Fisher Scientific StepOne called much lower Cq values, being up to 10 cycles lower than using the two other platforms. This difference is most probably due to the different default machine settings used to determine Cq. When the data are analyzed in RDML-Tools, the TD0 values, calculated from the same raw fluorescence data, are, as expected, very close to each other and machine and laboratory independent. The low SD values indicate the robustness of the algorithm calling the TD0 values.

In the same runs, described in Table 1, reactions using the same FSTL_1_*_259 amplicon but different amounts of human genomic DNA per reaction were included (Table 2).

Table 2 shows the results of a dilution series of input DNA. One would expect that, in case of an ideal PCR efficiency of 2, a Cq difference of one cycle would be found if the DNA concentration is 50% lower and 3.3 cycles if the concentration is 10-fold lower. Overall, the Cq and TD0 values reflect the expected difference in concentration in all three the machines. However, the range of Cq values is machine-dependent and the differences in Cq values between the dilutions are larger than expected with a PCR efficiency of 2.0, indicating that the PCR efficiency is lower. The same is observed for the TD0 values with the exception that the TD0 values are machine and laboratory independent.

### 2.2. PCR Efficiency Calculation

The PCR efficiency is the second indispensable factor that needs to be calculated from the amplification curve data. In RDML-Tools, the raw fluorescence data of each reaction are corrected for baseline background fluorescence which is derived from the individual amplification curve. The baseline background fluorescence value is calculated by linear regression of the exponential phase. In detail, this calculation works in two steps. The first step identifies the exponential phase by finding its end (stop cycle). The stop cycle is near the TD0, because close to TD0 the fluorescence still increases but its increase is not exponential anymore. Therefore, some distance is kept to the TD0, and the stop cycle is calculated by rounding down TD0—0.5. Next, the start cycle of the exponential phase is determined by going backwards from this stop cycle as long as the fluorescence decreases. The start cycle itself is excluded in the further calculations as it might be affected by residual baseline background. The exponential phase requires a minimum of 4 cycles and is limited to a maximum of 9 cycles.

At this point, the exponential phase is found but the baseline fluorescence has not been removed from the raw data, yet. The true baseline fluorescence is iteratively determined in the second step. In practice, the baseline fluorescence should be in the range between zero and the lowest fluorescence value of the exponential phase. Ideally, if the true baseline value was found and subtracted from the raw fluorescence measurements, the log transformed data points in the exponential phase should be on a straight line. This can be tested by splitting the log transformed values of the exponential phase into two subsections and by calculating the slope of each subsection. If the slope of the lower section is lower than that of the upper section, the baseline is underestimated. If it is the other way round, the baseline is overestimated. The true baseline is found by using the center between zero and the lowest fluorescence value in the exponential phase, applying it as a preliminary baseline and by testing the corrected fluorescence data for over- or underestimation of this baseline value. Then the center of the remaining range is used as baseline value and tested again. This calculation iterates until the difference between the slope of the lower section and the upper section is less than 0.00001. From the slope of the upper section the PCR efficiency is calculated by taking ten to the power of the slopes value and is recorded as the individual PCR efficiency of the analyzed reaction.

As the individual PCR efficiency is affected by measurement noise in the exponential phase, the mean PCR efficiency for each target in the run is calculated from the individual PCR efficiencies, reducing this variation. To avoid undue influence of a reaction with deviating PCR efficiency, RDML-RunAnalysis includes by default an iterative outlier test. If a skewed distribution of efficiency values is observed, the presence of an outlier is tested using a Grubbs test. If an outlier is found, the outlier is excluded from the analysis, the test is repeated and the mean PCR efficiency is recalculated [22].

The PCR efficiency calculation is similar to the one in the classic LinRegPCR software version 2021.2 [14] and old version of RDML-Tools v1.8.1 [22], except that in this new version of RDML-Tools no window of linearity is applied. The details of the implementation can be found in the linRegPCR function in rdml.py (https://github.com/RDML-consortium/rdmlpython). It is of relevance to note that the PCR efficiency calculation requires in contrast to the TD0 calculation an accurate baseline correction, which is only possible on raw fluorescence amplification data on which no baseline correction was applied.

### 2.3. Calculation of Ncopy

#### 2.3.1. Cq and N_0_ Are Not Intuitive Values

Cq values, whether or not efficiency corrected, are difficult to understand and counter intuitive. In both cases a higher Cq value means a lower DNA concentration and at some point, scientists start to do the conversion to number of copies in their head. The bias becomes even bigger when there is no information on the PCR efficiency, and the reader needs to assume a perfect PCR efficiency of 2 (100%) [13].

LinRegPCR introduced the N_0_ value, which is the fluorescence of the amplicon at the start of the PCR run. It had the advantage of being PCR efficiency corrected and a doubling of the N_0_ value corresponds to twice as much DNA in the reaction for a given amplicon. However, N_0_ values dependent on the measured fluorescence values and are as such PCR machine dependent, amplicon length dependent and due to their small value need to be expressed in scientific notation, which makes them less intuitive to understand.

This sparked already decades ago the idea of reporting the outcome of a PCR in the number of copies at the start of the reaction (Ncopy). The number of copies is very intuitive and easy to understand. Moreover, the investigator is immediately aware of the biological significance of the result and of possible issues of the Poisson effect when the Ncopy is below ten copies. Over the years many attempts were made to develop a reliable and correct conversion of the fluorescence values into Ncopy. Below we discuss various approaches we explored to reach this goal.

#### 2.3.2. Optical Calibrators

The simplest suggested approach to convert N_0_ values into Ncopy values would be to calibrate the fluorescence values measured by the PCR machine to a known DNA input in the reaction mix going through the PCR protocol without amplifying an amplicon [23,24]. We extensively tested this approach (Supplemental Data) using a known amount of DNA amplicon, plasmid DNA, lambda DNA or genomic DNA in a PCR mix including SYBR Green I, because this DNA binding dye is used as monitoring dye in most commercially available PCR master mixes. The measured fluorescence would directly reflect the amount of DNA in the mixture and as such could be used to convert N_0_ into Ncopy. When such a sample is included in a PCR experiment, one expects that the measured fluorescence signal should be the same in each cycle throughout the entire run and the input DNA could serve as an optical calibrator. Because optical calibrators go through the denaturation, annealing and elongation cycles, the DNA of the optical calibrator would need to melt and hybridize in each cycle completely and correctly to obtain a constant fluorescence value in consecutive cycles. However, we did not observe a constant fluorescence value when lambda DNA or sheared human genomic DNA was used. This is likely due to the fact that PCR reaction mix and the PCR protocol are not optimal for hybridization of complex DNA mixtures. Rehybridization of complex mixtures requires hybridization buffers with a high salt concentration, long hybridization times and hybridization temperatures below 60 °C. To avoid the issue of the complex DNA mixture, purified PCR amplicons were prepared. In principle this approach is working but only within a small window of DNA concentrations. The optical calibrators could only be reliably used in a range of 2 to 4 ng/µL. Above this range the fluorescence signal was not linear with the DNA input anymore, likely due to the limited amount of SYBR Green I in the master mix, and below this range the fluorescence signal was lost after a single round of denaturation, most probably because the DNA concentration is too low for efficient rehybridization. These data were obtained using the LightCycler 480 II, but could not be reproduced using the other machines. A likely possibility is that qPCR machines are not made to measure this type of signals. Moreover, the machine’s background correction, and normalization algorithms might interfere, making the measurements impossible. Taken together, this approach is not a reliable approach and was therefore not implemented in RDML-Tools to convert N_0_ values into Ncopy values.

#### 2.3.3. Ncopy Calculation Using a Limiting Component Approach

As shown before, the algorithm implemented in RDML-Tools allows reliable determination of the TD0 values of a given PCR, on different instruments and in different laboratories. It is obvious that the exponential phase ends because one of the components in the reaction mix becomes limiting and its concentration is reduced in such way that the PCR efficiency is reduced. If all primers would be used to form an amplicon, the number of copies of the target at the end of the PCR reaction (COPY) could be calculated using the molar primer concentration (PRIM), Avogadro constant (N_A_) of 6.022 × 10^23^ and the volume of the reaction mix in liters (VOL) (Equation (3)).

(3) $$COPY=PRIM\times N_{A}\times VOL$$

The amount of DNA of the amplicon in gram (AMOUNT) can be calculated using the molecular weight of a DNA base pair, being on average 660 g/mol, and the length of the amplicon (amp) (Equation (4)). A PCR of a 100 bp amplicon in a reaction mix of 20 µL with a primer concentration of 250 nM would produce 3 × 10^12^ copies or 330 ng of the amplicon when all primers are used.

(4) $$AMOUNT=PRIM\times amp\times VOL\times 660\;\frac{g}{bp\times mol\times L}$$

In practice not all primers end up in an amplicon and the efficiency is reduced before. Purification of the amplicon after the PCR and determining its amount using UV measurement revealed that up to 60% of the primers were used in the formation of amplicons (Supplemental Data). To correct for this, a primer correction constant (C_p_) needs to be introduced, giving the fraction of primers used when TD0 is called. We empirically determined this factor for hydrolysis probes as 0.1 and for saturating DNA binding dyes as 0.04, which are used as default values in RDML-Tools. The conversion to Ncopy requires besides the TD0 value also the mean PCR efficiency (E) of all reactions of the amplicon within the run (Equation (5)). Because the reaction volume is given for practical reasons in µL (vol) and the primer concentration in nM (prim), a conversion factor (10^15^) needs to be included in this equation to correct for the difference in these dimensions.

(5) $$Ncopy=\frac{C_{p}\times prim\times N_{A}\times vol}{10^{15}\times E^{TD0}}\;$$

SYBR Green I is a non-saturating dye and is a limiting factor in qPCR. The concentration of SYBR Green I (dye) in a given qPCR mix is almost never disclosed by the company. This issue is investigated in Section 2.4.5, from which we conclude to use 400 nM as the default value for the calculation in RDML-Tools. As SYBR Green I binds to the minor groove of a DNA helix, the length of the amplicon (amp) in bp needs to be included in the Ncopy calculation (Equation (6)), as well. Furthermore, we require a different empirically determined dye constant (C_d_), which is 1.5 for SYBR Green I (See Section 2.4.5).

(6) $$Ncopy=\frac{C_{d}\times dye\times N_{A}\times vol}{{amp\times 10}^{15}\times E^{TD0}}$$

Both equations (Equations (5) and (6)) can be used for quantifying double stranded DNA and assume double stranded DNA as input at the start of the PCR run. If single stranded DNA, like cDNA or synthetic amplicons, are used, Ncopy needs to be multiplied with a factor 2. The additional factor 2 is due to the fact that one molecule of single stranded cDNA will result after the first PCR cycle in one double stranded molecule of DNA, while one molecule of double stranded genomic DNA will result in two double stranded molecules of DNA. RDML-Tools expect by default single stranded DNA as input, but the user can indicate that double stranded DNA was used and the respective correction in the Ncopy calculation is applied.

To validate the Ncopy calculation, the experiment discussed above (Table 1 and Table 2) was further analyzed by converting the concentration of DNA into the expected number of target copies at the start of the experiment (Expected Copies) and comparing it to the Ncopy value determined by RDML-Tools. The reaction mix included SYBR Green I and therefore Equation (6) was used for the conversion. The data shown in Table 3 and Table 4 are derived from the same reactions as the data shown in Table 1 and Table 2, except that the amount of genomic DNA was converted to the expected copies, taking into account that FSTL1 is a single copy gene and no pseudogenes have been reported [21]. In Table 3 and Table 4 the Ncopy values are shown as determined by RDML-Tools using the raw fluorescence data of the runs performed on either of the three machines.

The Ncopy values for the different reactions using the QuantStudio and StepOne, which were ran in parallel in Heidelberg (Germany), are very close to the expected number of copies, the Ncopy values of the same reaction mixes ran on the LightCycler 480 II in Amsterdam (The Netherlands) were found to be approximately a quarter lower than the expected values of the different amounts of expected copies. The origin of this difference is further investigated in Section 2.4. It is worth noticing that in the conversion from TD0 to number of copies the TD0 value is the only parameter that changes while the mean PCR efficiency and the amplification limit do not change within one target. Therefore, even if the assumptions in this calculation are not completely accurate, Ncopy still reproduces the granular changes in TD0. Inaccuracies in the assumptions impact each result of one target as one common factor.

### 2.4. Evaluation of Ncopy in Practice

qPCRs were performed with different primer concentrations, amplicon length, reporter systems and commercial or home-made reaction mixes on different machines, to evaluate whether the Ncopy determination is insensitive for these differences and reports the expected values. A technical dataset reflecting this complexity was prepared, ran on the three different machines and analyzed using this new version of RDML-Tools. In the Section 2.4.1, Section 2.4.2, Section 2.4.3, Section 2.4.4, Section 2.4.5, Section 2.4.6, Section 2.4.7 and Section 2.4.8, the effect of the different parameters on the Ncopy determination are shown and discussed. The pipetting schemes, the used chemicals, PCR cycling protocols, raw fluorescence data and unprocessed results for each experiment are available in the Supplemental Data.

#### 2.4.1. Primers

A large number of primers pairs including a hydrolysis probes were designed using primerBLAST (https://www.ncbi.nlm.nih.gov/tools/primer-blast/, accessed on 21 August 2024) [25] to amplify the human Follistatin like 1 gene (FSTL1; genbank NM_007085.5). The amplicon sizes range from 40 to 820 bp and all primer sets were selected with a primer melting temperature of 60 ± 1 °C and a probe melting temperature of 65 ± 1 °C (Table 5 and Supplemental Data). The primer pair FSTL_1_*_259 was included as this one was published before [21] and was used for initial experiments. Six of these 19 amplicons (Table 5, grey rows) can also be evaluated using the hydrolysis probe. For all reactions, the cycling protocol started with a 10 min 95 °C step to activate the hot start polymerase and then followed by 45–70 cycles of 15 s at 95 °C, 20 s at 60 °C and 45 s at 72 °C. Afterwards a melting curve was recorded. This cycling protocol was used for most experiments and is also found in the respective RDML files (Supplemental Data).

**Figure 2:**
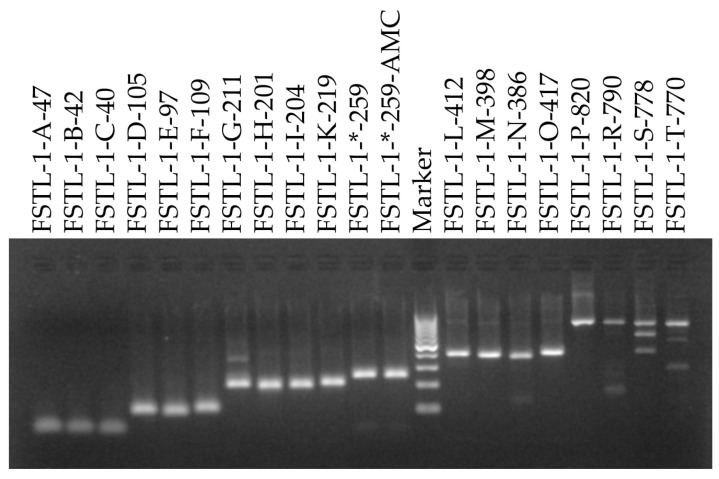
Picture of the agarose gel used to analyze the different FSTL-1 amplicons.

A reaction mixt containing 250 nM primers, 4 ng human genomic DNA (Human Genomic DNA; 11691112001, Roche Diagnostics GmbH, Mannheim, Germany) and the Roche SYBR Green I master mix (LightCycler 480 SYBR Green I Master; 04887352001, Roche Diagnostics GmbH, Mannheim, Germany) was prepared using seven technical replicates and was run on the QuantStudio 6. To check for the presence of artefacts, the melting curves were inspected and showed identical curves for the seven technical replicates (Supplemental Data). Subsequently, one of the technical replicates was loaded on an agarose gel. To obtain bands of equal intensity, the loaded amount was pipetted relative to the amplicon size (Figure 2). The gel analysis confirmed the melting curve analysis and primer pairs FSTL_1_G_211, FSTL_1_N_386, FSTL_1_R_790, FSTL_1_S_778 and FSTL_1_T_770 were removed from further analysis because artefacts were identified.

The raw fluorescence data were analyzed using RDML-Tools. The data suggest that shorter amplicons have a trend to higher PCR efficiencies (Table 6) and longer amplicons have a trend to lower TD0 values. Because FSTL1 is a single copy gene, there are 1200 copies of the FSTL1 gene expected in 4 ng human genomic DNA.

The calculated Ncopy values for each of the primer pairs were found to range from 593 to 1694. In all cases the standard deviation was relatively small, suggesting a reproducible determination. Although one would have expected that the differences in TD0 and PCR efficiency would compensate for each other in the calculation of Ncopy, this is not completely the case. The cause of these relative minute differences between the expected copies and Ncopy might reside in the remaining measurement noise after background correction in the individual measured fluorescence values and its impact on the calculation of the PCR efficiency.

#### 2.4.2. DNA

For studies aiming at absolute quantification and in particular for these Ncopy validation experiments, an accurately quantified double-stranded DNA target is required to calculate the expected copies and the number of copies present in the reaction (Ncopy). The PCR efficiency can be determined as the mean of the individual PCR efficiencies of all reactions analyzing the same target, but can also be derived from the slope of a DNA dilution series of the respective target. The disadvantage of the latter approach is that systematic errors in the dilution series will go unnoticed and bias the PCR efficiency calculation without increasing the SD [13]. Digital PCR (dPCR) allows to quantify the copy number of a target in a sample very accurately. The principle of dPCR is to separate the reaction into small droplets of nanoliter size and perform a PCR in each droplet [8,9,10]. After the PCR the droplets are analyzed for the presence of fluorescent signal. Analyzing thousands of these droplets, the target concentration in the sample can be calculated using Poisson statistics [26,27]. An advantage of this procedure is that the PCR efficiency hardly affects its outcome. A disadvantage of dPCR is that the amount of target input should be below 10^5^ copies per sample because of the maximal number of droplets that can be analyzed in a run and each droplet should ideally contain only one target.

**Figure 3:**
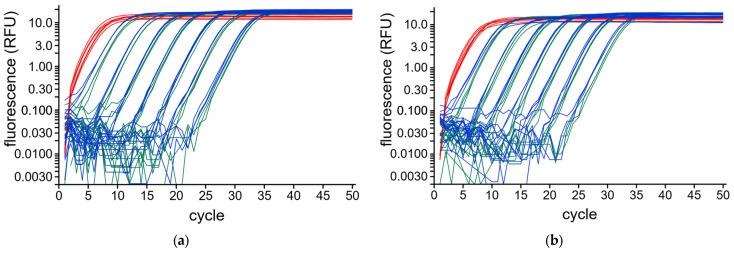
Baseline corrected amplification data of genomic DNA dilutions from 10^9^ to 100 copies: (**a**) Amplification using targets FSTL_1_H_201 (blue) and FSTL_1_L_412 (green); (**b**) Amplification using targets FSTL_1_F_109 (blue) and FSTL_1_K_219 (green). The baseline correction failed in the reactions with an input of 10^9^ copies (red).

To test the impact of DNA dilution over a broad range, a dilution series of a mix of two purified amplicons was created with freshly calibrated pipettes. A single 500 µL stock solution containing 125 ng FSTL_1_K_219 and 250 ng FSTL_1_L_412 being each 10^9^ copies per µL was prepared. The amplicon FSTL_1_L_412 can also be amplified using the internal primer pair FSTL_1_H_201 and amplicon FSTL_1_K_219 using the internal primer pair FSTL_1_F_109. The latter two can also be monitored with the hydrolysis probe. A dilution series reaching from 10^9^ down to 100 copies was created by serial 10-fold dilutions. The entire dilution series was amplified in triplicate using qPCR and analyzed using RDML-Tools to evaluate whether RDML-Tools are able to determine Ncopy over a broad range of input DNA. Figure 3 shows the background corrected amplification curves of the qPCR plotted on a logarithmic scale. The highest starting concentration (10^9^ copies) cannot be properly quantified because the exponential phase is too short for a correct baseline correction. Whereas all others are found in pairs at approximately 3 cycle intervals, reflecting the 10-fold dilution of the sample. The fact that the curve of the exponential phase of all reactions is parallel indicates a similar, if not the same PCR efficiency. This analysis showed that the amplicons of all four different targets could be precisely diluted over seven magnitudes and that the Ncopy values determined by RDML-Tools found the expected range from 100 up to 10^8^ copies.

**Figure 4:**
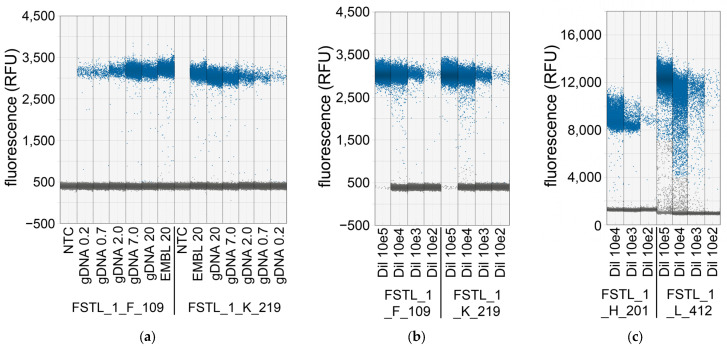
The panels show dPCR result plots of fluorescence vs. event: (**a**) Dilutions of the commercial genomic DNA; (**b**) Amplification of the amplicon dilution series using the hydrolysis probe; (**c**) Amplification of the amplicon dilution series using the DNA binding dye EvaGreen. Blue dots represent fluorescence positive droplets and grey blots fluorescence negative dots.

Commercial genomic DNA was used for the remaining experiments in this study, despite that genomic DNA is difficult to quantify exactly. Using UV-spectrometry (NanoDrop 1000, Thermo Fisher Scientific Inc., Santa Clara, CA, USA), fluorescent spectrometry (Q-bit) and qPCR, differences up to a factor 2 were found between the three different methods (Supplemental Data). To obtain the best possible insight, a dilution series of the commercial genomic DNA from 20 ng down to 0.2 ng per reaction was made and quantified by dPCR along with the dilutions 10^5^ to 100 copies from the amplicon dilution series described before using qPCR (Figure 3). A second aliquot of a different commercial DNA stock was included in an amount of 20 ng (EMBL 20). In the dPCR the hydrolysis probe was used as reporter in combination with the primer pairs FSTL_1_F_109 and FSTL_1_K_219, and the saturating dye EvaGreen, the commonly used DNA-binding dye in dPCR, was used in combination with the primer pairs FSTL_1_H_201 and FSTL_1_L_412. The results are summarized in Table 7 and Figure 4. The Ncopy results of the qPCR and the dPCR are comparable and show that the dilution series were made with high accuracy. This was further underscored by the observation that in both the qPCR and dPCR the 10-fold dilution steps were found back in the Ncopy values. While Ncopy for both analyzed amplicons are close to the expected copies over the entire dilution series, the Ncopy values found with dPCR were always higher than expected. Because of the absolute precision of dPCR, we concluded that the concentration of the commercial genomic DNA is higher than given by the manufacturer (Table 7). Based on this finding and conclusion, we used in our evaluations 1500 copies rather than 1200 copies as input when 4 ng of this commercial genomic DNA was used. Analyzing the purified amplicons as input revealed Ncopy numbers that are very similar using either qPCR or dPCR. These findings further substantiate that the RDML-Tools algorithm is robust and is able to calculate the expected values over the entire range of input amounts.

#### 2.4.3. Primer Concentration

The primer concentration is a critical parameter when setting up a qPCR reaction and should be carefully optimized. To evaluate whether the new version of RDML-Tools is able to correctly handle different primer concentrations, the primers were tested at a concentration of 100 nM, 250 nM and 750 nM. Of each condition seven replicates were included to obtain an accurate PCR efficiency determination. 4 ng human genomic DNA was used in combination with either the Roche SYBR Green I master mix or the SensiFast master mix (SensiFast SYBR No-Rox Mix; BIO-98005; Bioline GmbH; Luckenwalde; Germany) on the LightCycler 480 II machine (Table 8). As found in the dPCR experiment described before in Section 2.4.2, we used 1500 copies to represent the number of targets present is the used 4 ng commercial genomic DNA. Checking the PCR by melting curve analysis and agarose electrophorese, the expected amplicons were identified and did not show any artefacts for the majority of the primer pairs and concentrations.

The results summarized in Table 8 show a lot of difference between the determined Ncopy value and the expected 1500 copies in each reaction. However, due to the exponential nature of the amplification reaction, the PCR efficiency, which is the base in the kinetics formula (see Equation (1)) and TD0 the exponent, a small difference in PCR efficiency has a big effect on the calculated Ncopy value. To substantiate this, an error of 0.1 in the determined TD0 value and a PCR efficiency of 1.85, results in an error of a factor 0.06 in the calculated Ncopy value. On the other hand, a difference in PCR efficiency of 1.90 versus 1.85 results in a difference of a factor 2.1 in the calculated Ncopy value using a TD0 of 28. This consideration illustrates that the Ncopy calculation is limited by the precision of PCR efficiency estimation, which is in a similar range of 0.05 in this experiment (Table 8). In the past it was found that the effect of variation in the PCR efficiency of individual reactions could be largely reduced by using the mean PCR efficiency of all samples of the same target in one run [14]. It is important to note that RDML-Tools also uses the mean PCR efficiency of all reactions of a target for the calculation of Ncopy. As a consequence, all reactions of one target are affected by an identical, common factor and do not lose the minute differences found in the TD0 values. The reactions using the SensiFast master mix for all different amplicons have a lower TD0 values and higher PCR efficiency compared to the reactions using the Roche SYBR Green I master mix. This could be due to a difference in SYBR Green I concentration or a difference in the composition of the PCR master mix. Further optimization of the dye constant (C_d_) as used in Equation (6) could probably compensate for this effect. Moreover, the two largest amplicons have a determined Ncopy that better reflects the expected Ncopy value of 1500 using the SensiFast master mix. This might be the effect of a further optimized Taq-polymerase in the SensiFast master mix compared to the Roche SYBR Green I master mix in combination with the 45 s elongation time in the PCR cycles, which should be sufficient for synthesizing amplicons of 1500 to 4500 bp.

#### 2.4.4. PCR Efficiency Calculation Using DNA Dilutions

RDML-RunAnalysis calculates from each individual amplification curve the slope of the exponential phase, which is the default way in RDML-Tools, and reports the mean PCR efficiency for each different target within the run (Curve PCR eff). Alternatively, the PCR efficiency can be calculated by plotting the results of the dilutions as a calibration curve, fitting a straight line through the data and calculate the efficiency and the SD from the slope of the line. The evaluation of the calibration curve is implemented in RDML-ExperimentAnalysis and uses the determined individual TD0 values and the concentration of the DNA dilutions of the respective calibration samples for the calculation instead (Dilution PCR eff).

**Figure 5:**
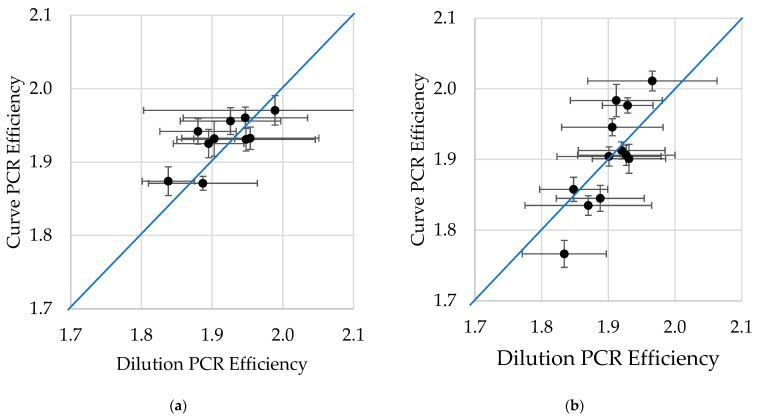
Comparison of the dilution PCR efficiency with the curve PCR efficiency using the Roche probe master mix (**a**) or the IDT probe master mix (**b**). The diagonal line represents the expected relation if both methods would result in the same efficiency values.

To compare both PCR efficiency determination methods, the previously dPCR validated genomic DNA dilution series including 20 ng, 7 ng, 2 ng, 0.7 ng and 0.2 ng was used with the different primer pairs at a concentration of 250 nM in combination with either the Roche SYBR Green I master mix or the SensiFast master mix (Supplemental Data). The curve PCR efficiency and the dilution PCR efficiency were plotted (Figure 5). Ideally, the same efficiency should be found with both methods and all values should be on a diagonal line. The dilution PCR efficiency has a higher SD than the curve PCR efficiency and the observed derivations from the ideal line are largely explained by the observed standard derivations.

At first glance the dilution PCR efficiency might look promising as it has a lower SD, but each of the two methods have their strength and weaknesses and, more important, offer an independent way to measure PCR efficiency. The curve PCR efficiency is determined in the same reaction as the amplification. If inhibitors or suboptimal conditions affect the amplification, the curve PCR efficiency is affected in the same way. Furthermore, it comes at no additional costs as no extra reactions are required on the plate and it can be calculated for any qPCR provided raw fluorescence data are available. The disadvantage of the curve PCR efficiency is its dependence on an accurate baseline correction and precise fluorescence measurement, which limit the resolution of this method as discussed in Section 2.4.3. Averaging the efficiencies of one target and including more reactions than in this experiment reduces SD on the calculated curve PCR efficiency. The dilution PCR efficiency approach has lower SD values, and the dilutions give in addition an indication of the linearity, the dynamic range and replicate variance of the assay. In practice, the dilution PCR efficiency approach has also several disadvantages. The dilutions require extra space for the reactions of each target in the plate. Moreover, and maybe of more importance is that the quality of the standard curve samples depends on the pipetting skills of the operator. Systematic dilution errors will go unnoticed as they will not increase the SD and result in a wrong dilution PCR efficiency. Last, diluting a DNA stock sample to obtain the different calibration curve samples does not only dilute the DNA but also dilutes potential inhibitors present in the stock sample, which can lead to overestimated dilution PCR efficiencies.

The curve PCR efficiency and the dilution PCR efficiency are calculated independent of each other and are affected by different components of the qPCR. Therefore, the confidence in the assay is enhanced when the results of both methods are in agreement. Furthermore, the comparison of both efficiencies and the evaluation of the calibration curve can help to trace problems in qPCR reactions when the results are not comparable.

#### 2.4.5. The SYBR Green Concentration

SYBR Green I is used as the monitoring dye in many qPCR mixes, for example in both the Roche SYBR Green I master mix and the SensiFast master mix used in this study. Most companies do not disclose the SYBR Green I concentration. An exception is ABP Biosciences, which sells a stock solution with a concentration of 200 µM (D010, ABP Biosciences, Rockville, MD, USA). Optical measurements of the absorbance at 494 nm on a NanoDrop 1000 revealed that this stock solution is similar to the SYBR Green I stock of Sigma Aldrich, if diluted 50-fold in DMSO (S9430, Sigma Aldrich, Darmstadt, Germany; Supplemental Data). The undiluted SYBR Green I stock from Sigma Aldrich would correspond to a 10 mM concentration, if an extinction coefficient (ε) of 60,000 is used, as suggested by Mao and coworkers [28]. Thus the 50-fold dilution of the Sigma Aldrich stock solution results in a SYBR Green I concentration of 200 µM in the measured solution. The Carl Roth SYBR Green I stock solution (1CN2.1, Carl Roth, Karlsruhe, Germany), which is sold at a concentration of 110 µM, reveals an unexpected absorbance reading when measured at 494 nm on the NanoDrop 1000 (Supplemental Data). The differences found in fluorescence can be explained by the differences in the used ε for the calculation of SYBR Green I concentration. ABP Biosciences indicates to use an ε of 66,000 while Carl Roth uses an ε of 27,000 (personal communication). As a consequence, the concentrations given by the companies cannot be used and the user must determine the optical density of the stock. Throughout the remainder of this study, we consider the SYBR Green I concentration of the ABP Biosciences stock solution to be 200 µM and related all dye concentrations in this study on this assumption. For convenience to other researcher willing to recreate our reaction mixes, ABP Bioscience stock solution of 200 µM results in an absorbance of 11.6 at 494 nm measured on the NanoDrop 1000.

SYBR Green I is used in a qPCR in non-saturating conditions, because a high concentration of SYBR Green I inhibit and even block amplification (Supplemental Data). To determine a suitable concentration for qPCR, three different qPCR master mixes that do not hold SYBR Green I, were supplemented with known amounts of SYBR Green I. The three mixes were two commercial qPCR probe mixes, the Roche Probe master mix (LightCycler^®^ 480 Probes Master; 04707494001; Roche Diagnostics GmbH, Mannheim, Germany) and the ITD Probe master mix (PrimeTime^®^ Gene Expression 2X Master Mix; 1055770; Integrated DNA Technologies, Leuven, Belgium), and the third mix we assembled ourselves (Supplemental Data). These three different reaction mixes were used as it has been reported that the composition of the reaction mix influences the effects of SYBR Green I. As a positive control the Roche SYBR Green I master mix and the SensiFast master mix were included. This analysis revealed that SYBR Green I concentrations above 0.5 µM impaired amplification. Comparison of the fluorescence levels of the mixes to the positive controls revealed that the SYBR Green I concentration is in the range of 0.2–0.5 µM (Supplemental Data) in the Roche SYBR Green I master mix and the SensiFast master mix. Based on these results, we have implemented 0.4 µM as default value for the SYBR Green I concentration and a C_d_ of 1.5 in the calculations of RDML Tools. If the accurate ε for SYBR Green I and its unambiguous concentration is reported, we will adapt these default values. Nevertheless, users themselves can always change these values in RDML-Edit in the dye section. This evident also hold if other values are found using another kit or different reaction mixes.

#### 2.4.6. EvaGreen

Saturating DNA-binding dyes, like LC-Green and EvaGreen, are less or not toxic to Taq polymerase. They can be used at concentrations at which they do not affect amplification and are in principle able to stain any amount of DNA in a reaction. Provided the company sells a qPCR master mix with a sufficient amount of the saturating dye, the primers are the limiting component and determine the end of the exponential phase. To investigate the limiting conditions of EvaGreen, EvaGreen (EvaGreen^®^ Dye—20X in Water; 31000-T; Biotium, Fremont, CA, USA) was added to the Roche Probe master mix (LightCycler^®^ 480 Probes Master; 04707494001; Roche Diagnostics GmbH, Mannheim, Germany) as well as the IDT probe master mix (PrimeTime^®^ Gene Expression 2X Master Mix; 1055770; Integrated DNA Technologies, Leuven, Belgium) in the generally used 1.25 µM concentration and tested with the different FSTL 1 primer pairs (Table 5) in combination with different amounts of input genomic DNA. The Roche probe master mix was tested with 7 ng, 2 ng and 0.7 ng of genomic DNA, while in the IDT probe master mix an additional fourth DNA input of 20 ng was included. All reactions were performed in triplicates. Although the experiments of the two probe master mixes were run on different days, the data are combined in Table 9. The reactions in which the primer pairs FSTL_1_K_219 and FSTL_1_L_412 were used revealed artefacts in the melting curve analysis independent of the used master mix and were omitted from the analysis. In this experiment the low number of reactions affected the calculation of the curve PCR efficiency to a large extent. As a consequence, the Ncopy values differ more from the expected values as in the experiments before. Moreover, this experiment also highlights the effect of different master mixes. Although the primer concentration is identical at 250 mM and the DNA concentrations are used from the same stock solutions, the Ncopy values obtained using the Roche Probe master mix are around four times lower than the Ncopy values using the IDT probe master mix. Therefore, we choose to use an average and determined the C_p_ constant of 0.04 as default value for saturating DNA-binding dyes, which was also used in Equation (5) for the calculation of Ncopy values shown in Table 9. At this point the C_p_ constant is not sufficiently precise for reactions using EvaGreen and more experiments are required to establish a more precise value for this constant. It might even be necessary to use different constants for different master mixes.

#### 2.4.7. Hydrolysis Probes

Using probes as monitoring chemistry rather than DNA-binding dyes has the advantage that probes are not affected by amplicon length as each amplicon will only bind one probe and artefacts are not included in the fluorescence measurements. The probe concentration has no concentration dependent effect on the detection, provided its concentration is lower than the primer concentration (Supplemental Data). This is likely due to its higher annealing temperature of 65 °C, which results in a stronger binding at the annealing temperature compared to the primers. Therefore, in the calculation of Ncopy the primer concentration is the limiting component rather than the concentration of the hydrolysis probe. To determine the C_p_ constant, five primer pairs were tested using two different mixes, the Roche Probe master mix and the IDT probe master mix. The two different master mixes resulted in slightly different Ncopy values (Table 10). The amplicon FSTL_1_T_770 did not amplify correctly using the Roche Probe master mix and was removed from the analysis. Based on this experiment and further experiments, where primer and probe concentration were varied (Supplemental Data), we determined the C_p_ constant to be 0.1 and set this value as default in RDML-Tools when using hydrolysis probes.

Using the IDT probe master mix revealed higher Ncopy values than using the Roche Probe master mix, despite that the primer and probe concentrations as well as the DNA input were identical. The Ncopy values found using the Roche probe master mix are close to the expected values. This finding suggests that this difference in Ncopy values is likely due to additional or different enhancers and stabilizers, like DMSO, present in both master mixes. Unfortunately, both companies do not disclose the composition of their master mixes for further investigation. As a consequence of this finding, the primer constant (C_p_) used in the calculations should be further optimized for the use of the IDT probe master mix.

#### 2.4.8. Comparison of Different Monitoring Chemistries

In a last experiment, the differences between the different monitoring chemistries were investigated. Three different stock mixes were prepared with 7 ng, 2 ng and 0.7 ng human genomic DNA per reaction, the Roche Probe master mix and the amplification primers at 250 nM each. After preparing these stock mixes, they were divided in three portions and the monitoring chemistry, SYBR Green I (250 nM), EvaGreen (1.25 µM) or the hydrolysis probe (150 µM), was added to each. From these final mixes three replicates were pipetted into the plate. This pipetting strategy aims at keeping all components as constant as possible between the different reactions. As a positive control and reference the same amounts of input DNA were also amplified using the Roche SYBR Green I master mix (Table 11). To calculate Ncopy in the reactions with the hydrolysis probe or EvaGreen as reporter, the primer limit approach given in Equation (5) was used, but with a different C_p_ of 0.1 and 0.04, respectively. When SYBR Green I or Roche SYBR Green I master mix was used, Ncopy was calculated using Equation (6), in which the dye is the limiting factor and 0.4 µM is the concentration of SYBR Green I (see Section 2.4.5). For all reactions an Ncopy value could be calculated and the Ncopy values are within a factor 3 range of the expected Ncopy value. Moreover, the 10-fold difference between the highest (7 ng) and lowest (0.7 ng) input is found back in the determined respective Ncopy values. The fact that the PCR efficiencies are within a close range, reflected by the small SD values, suggests that the monitoring chemistry does affect the performance of the qPCR. However, this conclusion is based on a relatively low number of reactions and as such, more experiments are required.

### 2.5. Comparison with Other qPCR Algorithms

In the early days of qPCR several algorithms for quantification were developed. Each algorithm has its strength and weakness. These algorithms were compared using a large data set, comprising 59 biomarkers and 5 reference genes evaluated in 366 different human patient cDNA samples and calibration curve samples using synthetic oligos to mimic each target [29,30]. This publication also includes a template to test future algorithms using this data set [30]. This template was converted to Python version 3.12.3 and included in RDML-Tools. In RDML-Tools an RDML-file is provided which includes the data set described above, allowing easy testing of newly developed procedures relative to the existing ones. The test.py routine recalculates the data as presented in the paper and more importantly also allows to include the results of an additional dataset, which was created using this new version of RDML-Tools for analysis. After analyzing the data, the results were compared to the other algorithms (Table 12). The analysis focuses on the different calibration curves samples which were created by synthetic oligo dilutions and their technical replicates. The table shows the bias, indicating the derivation of the observed values from the expected values, linearity, the distance of the mean of the observed values to the expected value of the regression line, precision, indicating the variance between technical replicates, and resolution, indicating the detectable difference based on a 95% confidence interval of the dilution series [30]. The rank of each method for each parameter is given in brackets, and the mean rank column is calculated based on these individual ranks. The low rank of the bias parameter is a consequence of a systematic dilution error of the standards in this dataset [13]. For this comparison, the intended, erroneous dilution was used to recreate the published results. In consequence, the dilution error of the standards affected the results of the bias parameter.

Taken together, this analysis shows that the new version of the RDML-Tools performs well and outcompetes most other tools. Using TD0 and removing the window of linearity calculation did not result in a lower analysis quality compared to the old version of RDML-Tools v.1.8.1 which is identical in performance to LinRegPCR version 2021.2.

### 2.6. Hints on Getting Ncopy Calculations to Work in Your Laboratory

Although the Ncopy calculations using RDML-Tools should work in any laboratory environment and RDML-Tools are intuitive to use, we suggest setting up the following pilot experiment to get familiar with the analysis pipeline. Perform a PCR experiment using your favorite qPCR master mix and PCR machine in which you determine Ncopy values using the FSTL_1_F_109, FSTL_1_H_201 and FSTL_1_*_259 primer pairs on a dilution series of human genomic DNA. The dilution series should encompass 20 ng, 7 ng, 2 ng, 0.7 ng, 0.2 ng and no human genomic DNA per reaction. Prepare three technical replicates of this dilution series. Run the following PCR program: 10 min 95 °C to activate the Taq polymerase followed by 50 cycles of 15 s at 95 °C, 20 s at 60 °C and 45 s at 72 °C. Retrieve the raw fluorescence data of the run from the qPCR machine and process with RDML-Tools, as described in detail in Section 2.7.

The result of this pilot experiment should be compared with the results provided in the Supplemental Data. The TD0 values should be within one cycle from the values given in Supplemental Data. The PCR efficiency might be different from those given in the supplemental data, but you should compare the dilution PCR efficiency with the curve PCR efficiency of your experiment using the quantify module in RDML-ExperimentAnaylsis. The efficiency calculation of both is expected to have some noise (±0.05), but both PCR efficiencies should be in statistical agreement in general. If lower curve PCR efficiency values than dilution PCR efficiency values are found, this most often points to issues with the baseline correction and are typical if machine corrected background data are used instead of raw data. This is further underscored when the same TD0 values but aberrant curve PCR efficiencies are found. The algorithm requires the use of raw data which are not baseline corrected, as this step is not reversable. Check the manual of your machine on how to retrieve the raw fluorescence values and re-run the RDML-Tools procedure. Next check the Ncopy values for the dilution series that should range from 6000 (20 ng) down to 60 (0.2 ng) copies of the target at the start of the reaction. If the Ncopy values using your favorite PCR master mix values are off, repeat the experiment but also include the Roche SYBR Green I master mix on your machine. If the difference remains, you might need to adapt the dye (C_d_) or primer constant (C_p_) for your reaction mix. Please contact us and let us know.

Below a step-by-step protocol is described to analyze qPCR data using RDML-Tools. The RDML-Help section provides a click-along tutorial for each tool.

### 2.7. Analyzing qPCR Data

All data in this publication were analyzed using an updated new version of the RDML-Tools [22], an open-source qPCR data analysis pipeline that, is available through https://www.gear-genomics.com/rdml-tools. This new version covers all steps, encompassing the import of fluorescence data into RDML-Tools, analysis of melting curves and amplification curves, calculation of Ncopy per reaction, inter-run correction, reference gene validation, normalization using reference genes, calibration of Ncopy using a single standard, between-group statistics and finally graphical presentation of the analysis results.

At this point it should be noted that the experimental design, the sampling of material and experimental procedures directly influence the quality and validity of the PCR outcome, and as such introduce technical and biological variation. Despite their effect on the outcome of a qPCR analysis, these sources of variation are not discussed here because they are not strictly affecting the analysis protocol; they are extensively reviewed elsewhere [31,32,33].

#### 2.7.1. Import of Raw Data

For a proper analysis, RDML-Tools requires raw data fluorescence data which are not baseline corrected by the PCR machine. You need to check the manual of your PCR machine on how to export the raw fluorescence data, because it is impossible to convert baseline corrected data back to raw fluorescence data. The export and subsequent import of raw fluorescence data is the most challenging part of the entire analysis as most machines have their own export format. The worldwide qPCR community made an appeal to all manufacturers [6], to make a raw fluorescence data output available in their machines in general and standardized file formats RDML [34] or RDES [6]. Today, many machines do not support these general formats and export all kinds of spreadsheets or text tables. As RDML-Tools can only import RDES files, the machine specific export file needs to be converted to the proper format using RDES-TableShaper. Note that in RDML-TableShaper the reformatting parameters for many machine formats are already present and ready for use. If your machine is not available, you can prepare it yourself and save the conversion file for future use. The details of this step are beyond the scope of this manuscript and are described step by step in the help section of RDML-Tools. If the conversion file of your machine is not available in the conversion formats provided, contact us so we can include this conversion format in the program.

#### 2.7.2. Getting the Data in Shape

In RDML-Tools many steps for the qPCR data analysis are automated provided the data are well annotated. The RDES format is used for the import of data and specifies the sample and target information per reaction. Note that reactions with the same sample name and target name are processed as technical replicates and all names are case sensitive. When biological samples from different experimental groups are included in the run, RDML-AnnotationEdit must be used to provide the sample grouping information that will be used in the RDML-ExperimentAnalysis. Per biological sample, the user can define one or more group annotations, like treatment, gender, age group, etc. The annotation is given in a key-value format and attached to the respective samples.

Besides details of targets and samples, also assay specifics need to be described. In RDML-Edit the reporter type should be selected on the dye tab. Information on the target should be provided as well, including the primer concentration, primer sequence and amplicon sequence and the expected melting temperature of the amplicon. Moreover, one needs to indicate whether the analyzed amplicon is a reference gene (ref) or a target of interest (toi). If there are standards among the samples, you should select the sample type “std” and provide the number of copies per µL in the reaction mix. Positive and negative controls should also be labeled with “pos” and “ntc”, respectively. Last, you should provide the reaction volume of the reaction on the experiment tab in RDML-RunEdit.

For advanced users who run the same analysis frequently, parts of the annotations like targets or samples can be copied between files using RDML-Merge. Once copied, RDML-RunEdit can connect reactions in that plate to the respective samples and targets.

#### 2.7.3. Melting Curve Analysis

DNA-binding dyes, like SYBR Green I and EvaGreen, do not discriminate between artefacts and the intended correct product. Because it is not possible to deduce the presence of an artifact in a reaction from the shape of its amplification curve [35], artefacts can in most cases be identified by analyzing the melting curve. In the melting curve analysis module of RDML-RunAnalysis, the correct product and artefacts are automatically detected, provided that the expected melting temperature of the intended targets is provided in the annotations. Reactions with artefacts are flagged and excluded from further analysis. Also, reactions in which the PCR has failed will be flagged. For an extensive description of the different flags, see our previous paper [22].

#### 2.7.4. Amplification Curve Analysis

The amplification curve analysis is at the heart of qPCR analysis and performs the calculations described in detail in this manuscript. It calls TD0 values, determines the PCR efficiency, calculates the Ncopy values and scores and flags deviating amplification curves. The user can evaluate the data on screen as the curves and associated values are visualized.

#### 2.7.5. Inter-Run Correction

Large biological experiments can be too big to fit all reactions in one PCR plate and thus the experiment must be spread over several plates. Although Ncopy values would be the exact number of target copies at the start of the reaction, its calculation is affected by small errors in the determination of the TD0 values, the PCR efficiency and baseline correction, as well as by the used plates, seals and detection of the fluorescence in the individual wells and affect the Ncopy calculation as a constant multiplicative factor [36,37].

RDML-Tools offers an inter-run correction that uses the Ncopy values of all reactions which overlap between the different plates of the entire experiment. Overlap is defined as biological and technical replicate reactions coming from the same experimental group in which the same target is amplified. This correction approach preserves the differences between targets and is robust for unbalanced designs in which some experimental groups are over- or underrepresented on the different plates in the experiment [36]. Most importantly, this approach has the advantage that a large number of different reactions are included in the estimation of the correction factor per plate which leads to cancelling out technical measurement error in individual reactions [36]. The inter-run correction procedure reports besides the corrected Ncopy values also the correction factors per run and thus allows the user to perform a quality check on runs that might have been noted to have problems during preparation of the run.

#### 2.7.6. Identification of Reference Genes

In order to cancel out variation between different biological samples the expression of genes of interest need to be normalized using a validated set of at least two reference genes. The latter are defined as targets of which the combined expression does not change in the studied experimental conditions [11]. As a consequence, reference genes should not be the usual suspects taken from literature because these are often not validated to be stable within the currently studied experimental conditions. It is, however, not trivial to find suitable reference genes. The best approach to identify suitable references genes is to select 10–12 genes of different biological pathways, and with different expression levels, from large data sources like RNA-seq data that are comparable to the planned experiments. The expression of these candidate reference genes is then measured in a pilot experiment including tissues from each of the different conditions (at least 8 samples) that are present in the planned experiment [11]. It is essential that in this pilot experiment all conditions are present once or balanced. The Ncopy results of the amplification curve analysis of this pilot experiment are then imported into the geNorm module of RDML-ExperimentAnalysis.

The implemented geNorm algorithm is an implementation of its original description [11] and its implementation in qBase plus program [38]. In short, the candidate reference genes are ranked based on their stability in the included samples. Thereafter, the algorithm determines how many of the most stable genes are required to form a sufficiently stable set of at least two reference genes. Note that this second step is critical; simply taking the two most stable genes might not be correct. When no set of candidate reference genes fulfills the stability criterion, a novel set of candidates needs to be selected, and the pilot experiment repeated. Once validate reference genes are identified they can be reused in every experiment evaluating the same experimental conditions.

#### 2.7.7. Quantification and Normalization

Initially, the technical replicates are handled by calculating the mean, standard deviation and the coefficient of variation (CV) per sample. This CV provides a quality measure for the differences between technical replicates. The user is notified when this CV is larger than 0.3, because in that case the technical variation adds more variation to the experiment than is to be expected.

Although RDML-RunAnalysis already reports Ncopy values based on the limiting condition at the end of the exponential phase, RDML-ExperimentAnalysis allows to scale the results based on a provided standard of known concentration. In this case the standard with the highest amount is used and all Ncopy values are multiplied by a correction factor that is calculated by dividing the provided, expected copies for this standard by its measured Ncopy value. In this case, when a single standard is used, it is advised to have at least 6 technical replicates and the number or targets in the reaction between 1000 and 5000 copies [13,18]. In a classic qPCR analysis, this kind of correction would correspond to absolute quantification.

RDML-RunAnalysis allows to combine this step with a normalization step. In the normalization, the Ncopy of each target of interest for each sample is divided by the geometric mean of the Ncopy of the reference gene expression in the same sample [11]. To avoid the loss of information on absolute expression of the genes of interest, RDML-ExperimentAnalysis multiplies this normalized expression by the overall geometric mean of all Ncopy values determined for all reference genes in all tissues in the experiment. This extension of the implementation of normalization will result in normalized Ncopy values of the target genes, allowing direct comparison of all genes of interest and experimental conditions in the entire experiment, without losing the intuitive absolute Ncopy values for each sample. In a classic qPCR analysis, this kind of correction would correspond to relative quantification.

#### 2.7.8. Experiment Statistics

In biological experiments with several treatment groups the Ncopy per sample is not the aim of the experiment. RDML-ExperimentAnalysis performs the statistical comparison between groups using the normalized Ncopy of individual samples per group which allows a comparison of the expression levels of the target genes across the different annotated experimental conditions or treatments. This test, after entering the required significance level (alpha), answers the question whether there is a significant difference between groups. In addition, the fold difference between a control and experimental group can be calculated using the mean of a treated group and defined control group. The fold difference between these two conditions is given with error bars, that are obtained by straight forward error propagation based on the SEM per group.

For comparison of two or more groups in a one-way design, parametric (*t*-test and one-way ANOVA) as well as non-parametric tests (Man-Whitney test and Kruskal-Wallis test) are implemented in RDML-ExperimentAnalysis. When, in a more-groups test, the null-hypothesis that all groups have an equal mean or median is rejected RDML-Tools will continue with a multiple comparison of groups within the context of the more-group test [39,40], thus avoiding the accumulation of Type 1 error when such multiple comparisons are carried out using separate two-sample tests for each combination. Note that the statistics, resulting from RDML-ExperimentAnalysis, should be regarded as a first evaluation of between-group differences. For more-way and repeated experimental designs, as well as regression analysis and other multivariate approaches, the user is referred to specialized statistical packages.

Finally, the results can be graphically displayed using RDML-BarGraph, which displays a box and whisker plot, in which the individual values are shown as dots.

## 3. Discussion

In this study we described the implementation and the experimental validation of a limited component approach to determine the number of copies of a target at the start of the reaction (Ncopy). The Ncopy calculation is based on three variables: The fractional number of cycles at which the end of the exponential phase is reached (TD0); the number of copies present when the exponential phase has ended and the PCR efficiency during the amplification.

We decided to use the term third derivative zero (TD0) instead of Cq to indicate that the quantification is based on a limiting condition at the end of the exponential phase and does not require the setting of a threshold. The TD0 value is identical to the second derivative maximum method, which was patented in 2001 [20] and used in Roche qPCR machines, but easier to determine. Our validation experiments showed that TD0 is more reproducible and machine independent compared to the threshold based Cq values calculated by the machine (Table 1 and Table 2). The superiority of the TD0 calculation is also reflected in the high rank of the precision in the algorithm comparison, which scores the difference between the technical replicates of a large dataset and compares the algorithm with ten other programs used to analyze qPCR data (Table 12).

The PCR efficiency can be calculated from the slope of the log-transformed and baseline corrected fluorescence measurements in the exponential phase (curve PCR eff) or from the calibration curve of a DNA dilution standard (dilution PCR eff). Both methods offer an independent approach to estimate PCR efficiency and should ideally result in the same PCR efficiencies. We found in our validation experiments that both methods are in agreement, although the calculated PCR efficiencies have a high SD. The precision of the PCR efficiency determination is affecting the calculation of Ncopy most. Nevertheless, the PCR efficiency should not be ignored and differences in PCR efficiency between templates are essential to be corrected especially when using reference genes and different genes of interest are compared.

In this new version of RDML-Tools, the individual TD0 and the mean PCR efficiency of the target in a plate are used to calculate Ncopy, the initial number of copies in the respective reaction. Ncopy has the advantage of being intuitively understandable. For the calculation, we discriminate only two cases, one when using SYBR Green I where the limit is depending on the length of the amplicon (Equation (6)) and the other case where it is not (Equation (5)). In the second case, primers or other components limit the amplification and are expressed in the factor of primers used at this point (C_p_). At this point it is relevant to realize that both equations can be combined and a primer concentration of 100 nM in Equation (5) would result in the same limit as an amplicon of 60 bp using a PCR master mix with 400 nM SYBR Green I in Equation (6). Ignoring additional effects that the reaction mix might have, amplicons shorter than 60 bp or primer concentrations below 100 mM will be limited by the primer concentration instead of the SYBR Green I dye. Our validation experiments showed that the Ncopy calculation is limited by the accuracy of the PCR efficiency calculation and as a consequence we were unable to detect this switch between the two conditions in our experiments. Therefore, we use in RDML-Tools Equation (6) for all non-saturating dyes and Equation (5) for all other reporters.

The validation of the Ncopy calculations under various conditions showed that the reported Ncopy values are usually within a range of a factor 3 of the expected number of copies using the default values. As this error affects all reactions of a target as a single, common factor, the minute differences between the reactions evaluating the same target are not lost. The experiments, especially using EvaGreen or hybridization probes, show that the used qPCR master mix affects the calculated Ncopy values. Therefore, if the C_p_ and C_d_ factors are adjusted to the mix used, the precision can possibly increase. The RDML-ExperimentAnalysis combines absolute and relative quantification in an intuitive way. While RDML-RunAnalysis reports Ncopy values, RDML-ExperimentAnalysis can use a single standard to scale the Ncopy values to its defined quantity. Subsequently, the Ncopy values can be corrected by the expression differences in reference genes when different biological samples are compared. As each step modifies the Ncopy values, the impact of the corrections is easy to understand and to evaluate.

For the evaluation of the limiting component method, we focused on determining limits of the assay and therefore aimed to reduce the effect of the input DNA by using a well-defined commercial stock and focusing on one, single copy gene. This controlled setup enabled a clear assessment of its performance, but it did not capture the variability introduced when amplifying different genes or complex biological samples [41,42]. Although we do not anticipate that this will be an issue, because the TD0 and PCR efficiency are determined from the raw fluorescence data of the individual amplification curve, we are looking forward to evolve this method with the scientific community.

We conclude that the Ncopy value is intuitive and independent of the machine, the master mix and monitoring chemistries used, provided the raw fluorescent data of the amplification run are exported and analyzed using this new version of RDML-Tools. Moreover, also qPCR data collected in the past can be reanalyzed as long as the raw fluorescence data and annotation data are available. Ncopy values can be directly compared between different targets and between different laboratories and countries.

## 4. Materials and Methods

This study used varied reaction mixes, fluorescence reporter systems, primer concentrations, amplicon length and more. For each qPCR run a pipetting scheme including the order information for the materials used, the raw fluorescence measurements in RDML format and the results of the analysis using RDML-Tools are available within the supplemental data on GitHub:

https://github.com/RDML-consortium/rdmlpython/tree/main/experiments/.

As the material is quite comprehensive, we created a file linking each reference in this article to the respective section in the supplemental data:

https://github.com/RDML-consortium/rdmlpython/tree/main/.

For this manuscript and the analysis of the experiments RDML-Tools version 3.0.1 was used. The RDML-Tools can be used from the EMBL server (https://www.gear-genomics.com/rdml-tools) or downloaded and installed (https://github.com/RDML-consortium/rdml-tools).

## Figures and Tables

**Table 1:** Comparison of Cq values automatically determined by the machine software (grey background) with TD0 values calculated by RDML-Tools. One large reaction mix was pipetted in different volumes into the plate. In the table the mean and the standard derivation of three technical replicates is given. Note that due to volume constraints the largest volume was not processed in the 384 well format and the smallest volume not in the 96 well format.

Volume (µL)	Machine Cq ± SD			RDML-Tools TD0 ± SD		
	LightCycler 480 II	QuantStudio 6	StepOne	LightCycler 480 II	QuantStudio 6	StepOne
	384 Wells	384 Wells	96 Wells	384 Wells	384 Wells	96 Wells
5	23.89 ± 0.04	23.34 ± 0.13		24.91 ± 0.03	24.82 ± 0.01	
10	23.93 ± 0.02	22.57 ± 0.09	16.84 ± 0.94	24.95 ± 0.04	24.78 ± 0.05	24.81 ± 0.03
20	24.17 ± 0.14	22.28 ± 0.03	13.65 ± 1.36	25.16 ± 0.14	24.78 ± 0.02	24.79 ± 0.04
40			11.61 ± 0.47			24.76 ± 0.03

**Table 2:** Reactions of different DNA concentrations were used to automatically determine the Cq values by the machine software (grey background) and the TD0 values by RDML-Tools. In the table the mean and the standard derivation of three technical replicates is given.

DNA (ng)	Machine Cq ± SD			RDML-Tools TD0 ± SD		
	LightCycler 480 II	QuantStudio 6	StepOne	LightCycler 480 II	QuantStudio 6	StepOne
	384 Wells	384 Wells	96 Wells	384 Wells	384 Wells	96 Wells
2	25.60 ± 0.06	24.68 ± 0.04		26.62 ± 0.11	26.62 ± 0.01	
4			16.53 ± 0.33			26.45 ± 0.08
10	23.36 ± 0.06	21.50 ± 0.17		24.34 ± 0.03	23.72 ± 0.03	
20	22.12 ± 0.01	20.53 ± 0.10	13.27 ± 0.46	23.14 ± 0.07	22.73 ± 0.01	23.76 ± 0.00
40	20.99 ± 0.03	19.46 ± 0.14	13.19 ± 0.41	22.00 ± 0.04	21.63 ± 0.01	22.70 ± 0.06
80			12.28 ± 0.84			21.64 ± 0.01

**Table 3:** One reaction mix was loaded in triplicate in different volumes into three reaction plates and processed on the different qPCR machines. The volume, the expected copies and the Ncopy values were calculated using the data of Table 1.

Volume (µL)	Expected Copies	RDML-Tools TD0 ± SD		
		LightCycler 480 II	QuantStudio 6	StepOne
		384 Wells	384 Wells	96 Wells
5	750	671 ± 12	768 ± 4	
10	1500	1313 ± 37	1572 ± 53	1505 ± 30
20	3000	2294 ± 201	3140 ± 39	3050 ± 77
40	6000			6238 ± 136

**Table 4:** Reactions with different amounts of genomic DNA were amplified in triplicate using three different qPCR machines. The DNA amount, the expected copies and the Ncopy values were calculated using the data of Table 2.

DNA (ng)	Expected Copies	RDML-Tools TD0 ± SD		
		LightCycler 480 II	QuantStudio 6	StepOne
		384 Wells	384 Wells	96 Wells
2	600	445 ± 33	479 ± 3	
4	1200			1049 ± 53
10	3000	1950 ± 40	3126 ± 62	
20	6000	4243 ± 180	5905 ± 51	5958 ± 17
40	12,000	8869 ± 229	12,030 ± 64	11,794 ± 429
80	24,000			23,402 ± 146

**Table 5:** Summary of the characteristics of all primer pairs used in this analysis. Primer pairs with grey background can be combined with the hydrolysis probe ACCCTTTCCCACCTGCAAAAGAGCCACCA (Tm 65.4 °C). It is relevant to note that FSTL1 is a single copy gene in the human genome with no reported pseudogenes.

	Amplicon Size	Forward Primer	Tm	Reverse Primer	Tm
FSTL_1_A_047	47	GGAAGACTAACTGAGAGAGGGGA	60.3	TTTGCAGGTGGGAAAGGGTT	60.0
FSTL_1_B_042	42	GGGGACTTAAACCCTTTCCCA	59.6	CACTGGTGGCTCTTTTGCAG	59.7
FSTL_1_C_040	40	TTGCTTAGGTGAGAGGAACCG	59.7	GGATCATTCTCTGACCTCTCGG	59.7
FSTL_1_D_105	105	AGGGAAGACTAACTGAGAGAGGG	60.3	AACAGGACAGAATTCCCATTTTGC	60.3
FSTL_1_E_097	97	AGTTGTGTTGGGTCAGTGTAGAG	60.2	TAAGCATCAGGACCACATACACC	60.1
FSTL_1_F_109	109	GTCAGAGAATGATCCACAAGGGA	59.8	CCCATTTTGCTTTTTACGTGACATT	59.5
FSTL_1_G_211	211	GGAGGAAGGGGGAAAATGCT	59.7	GGCATCCCACAAGCACAAAG	60.0
FSTL_1_H_201	201	CCTCACTTTGTGCTTGTGGG	59.3	ACTTTGGTGCCCGTAACTGA	59.5
FSTL_1_I_204	204	CAGCCTGGGTGATCTGACA	59.0	GTTAGCAAGACCTCAGCTTCG	59.0
FSTL_1_K_219	219	CGAGAGGTCAGAGAATGATCCAC	60.2	CTAGTCCTTGCAAGTTCTGACCA	60.2
FSTL_1_*_259	259	GGGAAAGGGTTTAAGTCCCC	57.7	TGGAGTAATGATGGAACAGGG	57.1
FSTL_1_L_412	412	AGGGAGCCTAGGGAAAGGAT	59.4	TCACTTTGGTGCCCGTAACT	59.5
FSTL_1_M_398	398	CTGGAGTCTTTGAGGGTTGCT	59.9	CCCAGATGGTGCAAGGGATT	60.0
FSTL_1_N_386	386	GGTGATTCTCGCCTGGACTC	60.2	TAACTTGCTCTGACGTGCCC	60.3
FSTL_1_O_417	417	AGGGAAGACTAACTGAGAGAGGG	60.3	ATGCCATAAAAGTAGACGGGCA	60.1
FSTL_1_P_820	820	GGCCCCCAGCCAAACTTATT	60.6	CCAGCCTCCTTGTCCTGTTC	60.3
FSTL_1_R_790	790	TGAGAGAGGGGACTTAAACCCT	59.9	TCCATCACTTCTTTCTTGGGCT	59.6
FSTL_1_S_778	778	GGAGGAAGGGGGAAAATGCT	59.7	CCTGGGACATGCAGAGAAGG	60.1
FSTL_1_T_770	770	GGAAGACTAACTGAGAGAGGGGA	60.3	CCCTGGCTCAATCACTTACTACT	59.6

**Table 6:** As an initial test the selected primer pairs were used to amplify 4 ng genomic DNA, equivalent to 1200 copies. The mean and standard deviation of the TD0, the mean PCR efficiency per amplicon and the Ncopy value for the seven technical replicates is given. The primer pair FSTL_1_*_259 was tested twice, once as newly synthesized primers together with the other primers and as an aliquot for the AMC stock.

	TD0 ± SD	PCR Eff ± SD	Ncopy ± SD
FSTL_1_A_047	26.88 ± 0.04	1.9972 ± 0.0315	646 ± 19
FSTL_1_B_042	26.87 ± 0.04	1.9354 ± 0.0303	1694 ± 46
FSTL_1_C_040	25.96 ± 0.05	2.0389 ± 0.0608	841 ± 30
FSTL_1_D_105	26.10 ± 0.02	1.9467 ± 0.0345	970 ± 15
FSTL_1_E_097	26.32 ± 0.05	1.9267 ± 0.0199	1185 ± 38
FSTL_1_F_109	26.44 ± 0.05	1.9334 ± 0.0246	890 ± 29
FSTL_1_H_201	26.08 ± 0.01	1.8844 ± 0.0575	1197 ± 10
FSTL_1_I_204	25.74 ± 0.05	1.8893 ± 0.0212	1370 ± 47
FSTL_1_K_219	25.62 ± 0.04	1.9011 ± 0.0124	1173 ± 27
FSTL_1_*_259	25.43 ± 0.07	1.9087 ± 0.0477	1010 ± 43
FSTL_1_*_259 AMC	25.55 ± 0.10	1.9387 ± 0.0382	630 ± 45
FSTL_1_L_412	25.79 ± 0.06	1.8687 ± 0.0351	873 ± 31
FSTL_1_M_398	25.65 ± 0.04	1.8706 ± 0.0151	958 ± 25
FSTL_1_O_417	25.66 ± 0.05	1.8842 ± 0.0191	756 ± 25
FSTL_1_P_820	26.24 ± 0.07	1.8278 ± 0.0054	593 ± 26

**Table 7:** Amplicon DNA dilutions (Dil) and commercial genomic DNA dilutions (gDNA) were quantified using qPCR and dPCR. The column marked with Expected copies gives the expected number of copies based on the amount of DNA used as input. The PCR efficiencies in qPCR were 1.896 for FSTL_1_F_109, 1.859 for FSTL_1_K_219, 1.855 for FSTL_1_H_201 and 1.848 for FSTL_1_L_412. The primer pairs FSTL_1_F_109 and FSTL_1_K_219 target the same region in the FSTL1 gene, as well as the primer pairs FSTL_1_F_201 and FSTL_1_L_421. For the amplicon dilutions (Dil) the mix of the purified amplicons FSTL_1_K_219 and FSTL_1_L_421 was used.

Sample	Expected Copies	Corrected Copies	qPCR		dPCR	qPCR		dPCR
			TD0 ± SD	Ncopy ± SD	Copies	TD0 ± SD	Ncopy ± SD	Copies
			FSTL_1_F_109			FSTL_1_K_219		
NTC					No Call			No Call
EMBL 20	6000	6000	24.73 ± 0.11	4458 ± 323	6094	23.55 ± 0.06	7477 ± 269	4349
gDNA 20	6000	7500	24.73 ± 0.07	4458 ± 209	7541	23.25 ± 0.16	9031 ± 871	7327
gDNA 7.0	2100	2700	26.40 ± 0.16	1535 ± 164	2793	25.02 ± 0.33	3046 ± 600	2520
gDNA 2.0	600	750	28.25 ± 0.09	470 ± 27	738	26.85 ± 0.10	963 ± 59	687
gDNA 0.7	210	270	29.99 ± 0.08	155 ± 8	256	28.81 ± 0.08	286 ± 15	270
gDNA 0.2	60	75	32.19 ± 0.15	38 ± 5	195	30.72 ± 0.25	88 ± 13	77
			FSTL_1_F_109			FSTL_1_K_219		
Dil 10e5	100,000	100,000	19.91 ± 0.05	97,713 ± 3180	104,858	19.16 ± 0.10	114,193 ± 7303	96,682
Dil 10e4	10,000	10,000	23.42 ± 0.06	10,316 ± 376	9803	22.83 ± 0.01	11,670 ± 64	9357
Dil 10e3	1000	1000	26.87 ± 0.03	1137 ± 19	1017	26.01 ± 0.16	1630 ± 159	975
Dil 10e2	100	100	30.67 ± 0.11	100 ± 7	88	29.73 ± 0.17	162 ± 17	88
			FSTL_1_H_201			FSTL_1_L_412		
Dil 10e5	100,000	100,000	19.55 ± 0.13	101,901 ± 7770	NA	19.04 ± 0.07	73,314 ± 3104	42,967
Dil 10e4	10,000	10,000	23.36 ± 0.35	9812 ± 1999	10,222	23.00 ± 0.12	6456 ± 452	6686
Dil 10e3	1000	1000	26.52 ± 0.02	1377 ± 17	1107	25.97 ± 0.16	1044 ± 102	850
Dil 10e2	100	100	30.41 ± 0.30	126 ± 22	86	29.76 ± 0.31	103 ± 19	70

**Table 8:** The Roche SYBR Green I master mix and the SensiFast master mix were used with 4 ng commercial genomic DNA, equivalent to 1500 copies, using different primer pairs in three different concentrations. Of all different combinations seven technical replicates were amplified. Reaction results with a grey background showed artefacts in the melting curves, invalidating the reported values.

	Roche SYBR Green I Mix			SensiFast Mix		
	Primer Concentration: 100 mM			Primer Concentration: 100 mM		
	TD0 ± SD	PCR eff ± SD	Ncopy ± SD	TD0 ± SD	PCR eff ± SD	Ncopy ± SD
FSTL_1_A_047	27.28 ± 0.12	1.8814 ± 0.1195	2513 ± 190	26.22 ± 0.12	1.9594 ± 0.0338	1692 ± 135
FSTL_1_B_042	27.09 ± 0.13	1.9014 ± 0.2566	2377 ± 189	26.52 ± 0.09	1.8845 ± 0.0954	4330 ± 250
FSTL_1_D_105	27.73 ± 0.06	1.8191 ± 0.0487	2148 ± 71	26.13 ± 0.04	1.9007 ± 0.0411	1779 ± 50
FSTL_1_E_097	27.77 ± 0.05	1.8715 ± 0.0850	1031 ± 33	26.23 ± 0.13	1.8635 ± 0.0827	3034 ± 232
FSTL_1_F_109	29.86 ± 0.10	1.8034 ± 0.0576	749 ± 44	27.32 ± 0.06	1.9213 ± 0.1344	592 ± 22
FSTL_1_H_201	28.38 ± 0.16	1.8482 ± 0.0426	485 ± 46	26.85 ± 0.05	1.8773 ± 0.0696	815 ± 25
FSTL_1_I_204	29.62 ± 0.11	1.7883 ± 0.0362	591 ± 36	26.94 ± 0.07	1.8655 ± 0.0485	898 ± 38
FSTL_1_K_219	29.02 ± 0.08	1.7714 ± 0.0500	1025 ± 47	25.75 ± 0.08	1.8526 ± 0.0708	2099 ± 103
FSTL_1_*_259	27.33 ± 0.15	1.8720 ± 0.0951	505 ± 46	25.67 ± 0.06	1.8631 ± 0.0563	1611 ± 62
FSTL_1_L_412	28.54 ± 0.16	1.7647 ± 0.0528	804 ± 72	27.16 ± 0.08	1.8047 ± 0.0465	955 ± 46
FSTL_1_M_398	33.44 ± 0.63	1.7578 ± 0.0717	61 ± 17	25.38 ± 0.13	1.8856 ± 0.0542	932 ± 74
FSTL_1_P_820	34.48 ± 0.28	1.7450 ± 0.0566	21 ± 3	25.84 ± 0.06	1.8308 ± 0.0402	721 ± 27
	Primer Concentration: 250 mM			Primer Concentration: 250 mM		
	TD0 ± SD	PCR eff ± SD	Ncopy ± SD	TD0 ± SD	PCR eff ± SD	Ncopy ± SD
FSTL_1_A_047	26.69 ± 0.05	1.9603 ± 0.0606	1213 ± 39	25.84 ± 0.04	1.9391 ± 0.1113	2844 ± 78
FSTL_1_B_042	26.71 ± 0.06	2.0191 ± 0.1042	609 ± 26	26.41 ± 0.10	1.9264 ± 0.0756	2600 ± 177
FSTL_1_D_105	26.16 ± 0.07	2.0076 ± 0.0807	417 ± 20	25.41 ± 0.10	1.9194 ± 0.0758	2203 ± 150
FSTL_1_E_097	26.47 ± 0.09	1.9105 ± 0.0471	1347 ± 75	25.63 ± 0.05	1.8916 ± 0.0212	2992 ± 106
FSTL_1_F_109	26.63 ± 0.05	1.8878 ± 0.0859	1485 ± 49	25.66 ± 0.06	1.8585 ± 0.0405	4105 ± 154
FSTL_1_H_201	26.00 ± 0.06	1.8845 ± 0.0469	1261 ± 47	25.50 ± 0.09	1.8453 ± 0.0329	2960 ± 162
FSTL_1_I_204	26.10 ± 0.08	1.9255 ± 0.0665	662 ± 35	24.98 ± 0.04	1.8702 ± 0.0390	2864 ± 67
FSTL_1_K_219	25.78 ± 0.07	1.8833 ± 0.0496	1350 ± 57	24.63 ± 0.05	1.9396 ± 0.0764	1355 ± 45
FSTL_1_*_259	25.27 ± 0.11	1.8848 ± 0.0599	1543 ± 107	24.69 ± 0.07	1.8901 ± 0.0455	2087 ± 94
FSTL_1_L_412	25.86 ± 0.05	1.9167 ± 0.0183	434 ± 13	25.05 ± 0.07	1.8543 ± 0.0675	1683 ± 69
FSTL_1_M_398	30.90 ± 0.87	1.8173 ± 0.0795	95 ± 33	24.59 ± 0.06	1.8496 ± 0.0456	2457 ± 92
FSTL_1_P_820	32.30 ± 0.30	1.8215 ± 0.0646	17 ± 3	25.03 ± 0.04	1.8473 ± 0.0541	938 ± 26
	Primer Concentration: 750 mM			Primer Concentration: 750 mM		
	TD0 ± SD	PCR eff ± SD	Ncopy ± SD	TD0 ± SD	PCR eff ± SD	Ncopy ± SD
FSTL_1_A_047	26.65 ± 0.06	1.9316 ± 0.1051	1847 ± 72	24.61 ± 0.07	1.8278 ± 0.0468	27,519 ± 1223
FSTL_1_B_042	26.69 ± 0.03	1.9779 ± 0.0631	1070 ± 20	25.21 ± 0.07	1.8936 ± 0.0658	8796 ± 418
FSTL_1_D_105	25.89 ± 0.05	1.9745 ± 0.0797	771 ± 28	24.62 ± 0.04	1.8963 ± 0.0427	4945 ± 126
FSTL_1_E_097	26.19 ± 0.07	1.9079 ± 0.0666	1669 ± 72	25.09 ± 0.07	1.9022 ± 0.0556	3682 ± 170
FSTL_1_F_109	26.00 ± 0.04	1.9121 ± 0.0405	1593 ± 37	25.35 ± 0.10	1.9227 ± 0.1173	2111 ± 132
FSTL_1_H_201	25.57 ± 0.05	1.9097 ± 0.0538	1179 ± 39	24.84 ± 0.05	1.8978 ± 0.0433	2198 ± 65
FSTL_1_I_204	25.44 ± 0.14	1.8745 ± 0.0279	2027 ± 186	24.43 ± 0.15	1.8517 ± 0.0537	5156 ± 462
FSTL_1_K_219	25.37 ± 0.10	1.9944 ± 0.1479	410 ± 29	24.50 ± 0.06	1.9511 ± 0.1175	1279 ± 53
FSTL_1_*_259	25.04 ± 0.11	1.9383 ± 0.0521	889 ± 67	24.35 ± 0.07	1.9514 ± 0.0678	1187 ± 55
FSTL_1_L_412	25.14 ± 0.05	1.8768 ± 0.0730	1172 ± 38	24.00 ± 0.04	1.8607 ± 0.0455	2959 ± 64
FSTL_1_M_398	29.35 ± 0.21	1.8405 ± 0.0835	153 ± 20	24.18 ± 0.06	1.9155 ± 0.0540	1357 ± 57
FSTL_1_P_820	31.48 ± 0.32	1.8485 ± 0.0824	18 ± 3	24.60 ± 0.09	1.8113 ± 0.0262	1988 ± 109

**Table 9:** Different amounts of genomic DNA were amplified using different primer pairs and the Roche Probe master mix or the IDT probe master mix, both complemented with a final concentration of 1.25 µM EvaGreen. For each different combination three technical replicates were evaluated.

Target	Curve PCR Eff (SD)	Ncopy ± SD	Ncopy ± SD	Ncopy ± SD	Ncopy ± SD
Expected		7500	2600	750	260
	Roche Probe Mix				
FSTL_1_A_047	2.029 ± 0.033		602 ± 32	143 ± 9	44 ± 1
FSTL_1_B_042	1.979 ± 0.065		735 ± 19	173 ± 26	65 ± 5
FSTL_1_D_105	1.960 ± 0.051		1100 ± 53	305 ± 6	112 ± 7
FSTL_1_E_097	1.989 ± 0.051		1247 ± 67	291 ± 6	92 ± 10
FSTL_1_F_109	1.967 ± 0.018		944 ± 44	271 ± 14	89 ± 1
FSTL_1_H_201	1.895 ± 0.016		2531 ± 228	605 ± 87	194 ± 29
FSTL_1_I_204	1.968 ± 0.041		1379 ± 94	285 ± 13	90 ± 4
FSTL_1_M_398	1.923 ± 0.034		1475 ± 404	373 ± 67	105 ± 14
	IDT Probe Mix				
FSTL_1_A_047	1.848 ± 0.073	24,181 ± 404	9055 ± 109	2749 ± 33	1056 ± 56
FSTL_1_B_042	1.852 ± 0.039	18,255 ± 451	7109 ± 33	2150 ± 67	798 ± 37
FSTL_1_D_105	1.885 ± 0.018	11,562 ± 185	4393 ± 77	1253 ± 22	416 ± 9
FSTL_1_E_097	1.896 ± 0.012	11,369 ± 181	4155 ± 70	1167 ± 54	413 ± 11
FSTL_1_F_109	1.873 ± 0.027	12,157 ± 228	4741 ± 28	1323 ± 30	454 ± 24
FSTL_1_H_201	1.820 ± 0.015	16,666 ± 1521	5426 ± 771	1526 ± 38	507 ± 5
FSTL_1_I_204	1.871 ± 0.014	14,647 ± 4180	6105 ± 71	1674 ± 63	521 ± 27
FSTL_1_M_398	1.863 ± 0.009	16,750 ± 285	6053 ± 57	1588 ± 98	508 ± 26

**Table 10:** Different amounts of genomic DNA were amplified using the different primer pairs which are compatible with the hydrolysis probe (see Table 5) and either the Roche Probe master mix or the IDT probe master mix. All different combinations were run in triplicate.

Target	Curve PCR Eff ± SD	Ncopy Max ± SD	Ncopy Min ± SD	Ncopy Min ± SD	Ncopy Min ± SD	Ncopy Ratio ± SD
Expected		7500	2700	750	270	75
	Roche Probe Mix					
FSTL_1_D_105	1.944 ± 0.097	7206 ± 302	2814 ± 26	770 ± 40	278.9 ± 12.6	83.1 ± 5.3
FSTL_1_F_109	1.947 ± 0.081	6197 ± 281	2472 ± 78	645 ± 26	210.5 ± 11.8	71.0 ± 8.0
FSTL_1_K_219	1.943 ± 0.094	6247 ± 260	2390 ± 73	662 ± 34	244.5 ± 27.9	56.7 ± 7.2
FSTL_1_O_417	1.914 ± 0.060	4688 ± 379	1909 ± 327	578 ± 48	193.7 ± 18.2	56.1 ± 14.4
	IDT Probe Mix					
FSTL_1_D_105	1.923 ± 0.064	8404 ± 151	3583 ± 65	1010 ± 22	321.0 ± 8.1	119.1 ± 11.7
FSTL_1_F_109	1.909 ± 0.041	10,108 ± 727	3425 ± 39	1009 ± 38	341.1 ± 8.3	111.2 ± 17.7
FSTL_1_K_219	1.873 ± 0.035	15,691 ± 924	5889 ± 130	1755 ± 111	648.5 ± 31.9	218.6 ± 51.2
FSTL_1_O_417	1.909 ± 0.120	8548 ± 149	3021 ± 343	817 ± 37	310.6 ± 14.2	93.7 ± 3.8
FSTL_1_T_770	1.854 ± 0.067	11,307 ± 1595	3574 ± 429	914 ± 25	338.7 ± 52.9	89.2 ± 3.9

**Table 11:** Different reporters added to the Roche Probe master mix and tested with three DNA amounts in combination with two primer pairs and the indicated reporter system. For comparison, the Roche SYBR Green I master mix was included as well. Of each combination three technical replicates were processed.

Target	Reporter	Curve PCR Eff ± SD	Ncopy ± SD	Ncopy ± SD	Ncopy ± SD
Expected			2600	750	260
FSTL_1_D_105	Hydrolysis Probe	1.923 ± 0.060	1722 ± 79	539 ± 59	179.7 ± 32.6
FSTL_1_D_105	Eva Green	1.960 ± 0.051	1100 ± 53	305 ± 6	111.6 ± 7.2
FSTL_1_D_105	SYBR Green I	1.980 ± 0.037	1258 ± 32	314 ± 11	111.2 ± 6.3
FSTL_1_D_105	Roche SYBR Green I Mix	1.944 ± 0.020	2345 ± 6	659 ± 18	193.9 ± 9.4
FSTL_1_F_109	Hydrolysis Probe	1.943 ± 0.035	1265 ± 58	327 ± 14	102.0 ± 0.5
FSTL_1_F_109	Eva Green	1.967 ± 0.018	944 ± 44	271 ± 14	89.4 ± 1.5
FSTL_1_F_109	SYBR Green I	1.997 ± 0.041	988 ± 34	263 ± 8	81.8 ± 1.8
FSTL_1_F_109	Roche SYBR Green I Mix	1.942 ± 0.062	1980 ± 52	530 ± 9	164.3 ± 7.0

**Table 12:** Comparison of different qPCR tools.

	Bias	Linearity	Precision	Resolution	Mean Rank
**RDML-Tools v3.0.1**	**5.60 (4)**	**3.65 (2)**	**2.97 (1)**	**2.76 (1)**	**2.00**
Cy0	2.06 (2)	3.21 (1)	3.76 (3)	3.38 (3)	2.25
Standard-Cq	2.00 (1)	4.51 (4)	4.44 (4)	4.33 (4)	3.25
LinRegPCR	7.46 (8)	4.14 (3)	3.06 (2)	3.16 (2)	3.62
MAK2	5.51 (3)	5.32 (6)	5.62 (6)	5.56 (6)	5.25
PCR-Miner	6.52 (6)	4.79 (5)	5.25 (5)	5.03 (5)	5.25
LRE-E100	5.75 (5)	5.49 (7)	5.90 (7)	5.78 (7)	6.50
5PSM	9.84 (12)	7.19 (8)	7.08 (8)	7.35 (8)	9.00
DART	9.35 (11)	8.84 (9)	8.91 (9)	9.14 (9)	9.50
4PLM	8.70 (10)	9.52 (10)	9.79 (10)	9.67 (10)	10.00
LRE-Emax	7.75 (9)	10.13 (11)	10.45 (11)	10.52 (11)	10.50
FPK-PCR	7.46 (8)	11.21 (12)	10.75 (12)	11.32 (12)	10.88

## Data Availability

All data created for this study are available in the GitHub repository: https://github.com/RDML-consortium/rdmlpython/tree/main/experiments/untergasser.

## Supplementary Materials

The following supporting information can be downloaded at: https://github.com/RDML-consortium/rdmlpython/tree/main/experiments/ and others mentioned in Materials and Methods section.
